# Dynamic hub load predicts cognitive decline after resective neurosurgery

**DOI:** 10.1038/srep42117

**Published:** 2017-02-07

**Authors:** Ellen W. S. Carbo, Arjan Hillebrand, Edwin van Dellen, Prejaas Tewarie, Philip C. de Witt Hamer, Johannes C. Baayen, Martin Klein, Jeroen J. G. Geurts, Jaap C. Reijneveld, Cornelis J. Stam, Linda Douw

**Affiliations:** 1 Department of Anatomy & Neurosciences, VU University Medical Center, Amsterdam Neuroscience, Amsterdam, The Netherlands; 2 Department of Clinical Neurophysiology and MEG Center, VU University Medical Center, Amsterdam, The Netherlands; 3 Department of Psychiatry, University Medical Center Utrecht, Utrecht, The Netherlands; 4 Brain Center Rudolf Magnus, Utrecht, The Netherlands; 5 Department of Neurology, Neuroscience Campus Amsterdam, VU University Medical Center, Amsterdam, The Netherlands; 6 Sir Peter Mansfield Imaging Centre, School of Physics, University of Nottingham, Nottingham, UK; 7 Department of Neurosurgery, Amsterdam Neuroscience, VU University Medical Center, Amsterdam, The Netherlands; 8 VUmc CCA Brain Tumor Center Amsterdam, Amsterdam, The Netherlands; 9 Department of Medical Psychology, VU University Medical Center, Amsterdam, The Netherlands; 10 Athinoula A. Martinos Center for Biomedical Imaging, Massachusetts General Hospital, Charlestown, MA, USA

## Abstract

Resective neurosurgery carries the risk of postoperative cognitive deterioration. The concept of ‘hub (over)load’, caused by (over)use of the most important brain regions, has been theoretically postulated in relation to symptomatology and neurological disease course, but lacks experimental confirmation. We investigated functional hub load and postsurgical cognitive deterioration in patients undergoing lesion resection. Patients (n = 28) underwent resting-state magnetoencephalography and neuropsychological assessments preoperatively and 1-year after lesion resection. We calculated stationary hub load score (SHub) indicating to what extent brain regions linked different subsystems; high SHub indicates larger processing pressure on hub regions. Dynamic hub load score (DHub) assessed its variability over time; low values, particularly in combination with high SHub values, indicate increased load, because of consistently high usage of hub regions. Hypothetically, increased SHub and decreased DHub relate to hub overload and thus poorer/deteriorating cognition. Between time points, deteriorating verbal memory performance correlated with decreasing upper alpha DHub. Moreover, preoperatively low DHub values accurately predicted declining verbal memory performance. In summary, dynamic hub load relates to cognitive functioning in patients undergoing lesion resection: postoperative cognitive decline can be tracked and even predicted using dynamic hub load, suggesting it may be used as a prognostic marker for tailored treatment planning.

Resective surgery is a common treatment modality for patients with intracranial lesions, such as brain tumors or mesial temporal sclerosis (MTS). However, neurosurgery carries the risk of postoperative cognitive deterioration. Lesion and patient-related factors, such as lesion type and location, are only partially predictive^1^,^2^,^3^,^4^. We propose an improved method of predicting cognitive deterioration in the individual patient. The functional brain network represents patterns of synchronized activity between distinct brain regions, and can be reconstructed with magnetoencephalography (MEG) or resting-state functional MRI (rsfMRI). It is known that functional network topology relates to cognitive functioning in lesion resection candidates^5^,^6^,^7^,^8^. Moreover, network normalization after lesion resection relates to good cognitive outcome^9^,^10^. However, it is unclear which specific features of the network may be used to predict individual cognitive outcome.

The most important nodes in a network, ‘hubs’, seem seminal to take into account. These regions process a large proportion of total information flow, thereby being theoretically^11^,^12^ and experimentally linked to brain functioning: hub properties correlate with cognitive functioning in lesion resection candidates^9^,^10^,^13^,^14^. Moreover, hub load predicts cognitive outcome in traumatic brain injury patients^15^.

In addition, the temporal evolution of network patterns has recently gained much attention: the functional brain network is not a stationary entity, but is characterized by dynamically changing patterns of functional connectivity^16^,^17^,^18^,^19^. Importantly, the dynamics of connectivity yield better explanation of healthy cognitive variance than stationary properties^20^, with increased dynamics presumed important for distribution of cognitive demands while preventing node overload. One may therefore hypothesize that having hubs that are less dynamic puts more stress on the network, correlating with poorer cognition. Indeed, MTS patients with memory impairments have decreased dynamic hub connectivity (measured with rsfMRI) compared to patients without memory disturbances^21^.

We expect that increased stationary hub load and/or decreased dynamic hub load relates to poorer cognitive functioning before resection. Moreover, we hypothesize that hub (over)load is able to track and predict postoperative cognitive deterioration.

## Materials and Methods

### Patients

All patients undergoing lesion resection (predominantly brain tumors) at the Neurosurgical Center Amsterdam between 2009 and 2014 were eligible for participation. Part of these patient data has previously been described^8^,^9^. Inclusion criteria were (1) age over 17 years, (2) presence of a brain lesion and at least one epileptic seizure, (3) ability to complete neuropsychological testing, and (4) clinically and radiologically stable disease during follow-up. All patients gave written informed consent before participation. This study was approved by the medical ethics committee of the VU University Medical Center.

Two time points were analyzed: approximately 3 months before resection (PRE) and approximately one year after (POST). Each consisted of resting-state MEG and neuropsychological assessment, which was complete in 28 patients (see results section). Additionally, 28 subjects were chosen from a previously described healthy control group^22^, individually matched with respect to age, gender, and education. All methods were carried out in accordance with relevant guidelines and regulations (STROBE).

### Neuropsychological assessment

Individual cognitive performance measurement was determined using methodology employed in previous studies (see Supplementary Methods). Subtest z-scores were calculated using data from a matched, normative sample. These z-scores were then averaged into cognitive domains previously reported in comparable patient populations^9^,^23^, namely verbal memory, attention, and executive functioning. Unfortunately, we did not have similar cognitive scores available for the same controls that underwent MEG recordings, precluding us from investigating the association between cognition and SHub/DHub in healthy controls.

### Magnetoencephalography and connectivity analysis

For MEG recording and processing, we used methodology used in previous studies^8^,^9^,^24^. Five minute resting-state recordings took place in a magnetically shielded room (VacuumSchmelze GmbH, Hanua, Germany) using a 306-channel whole-head neuromagnetometer (Elekta Neuromag Oy, Helsinki, Finland). The MEG recording was co-registered to patients’ anatomical T1 MRI, to which the automated anatomical labeling (AAL) atlas was also transformed. A scalar beamformer (Elekta Neuromag Oy, beamformer, version 2.1.28) was used to estimate neurophysiological activity in each of the 78 cortical regions in the atlas^25^. This yielded a number of time series (epochs) per subject, each 2048 samples (1.638 s per epoch, 65.5 s in total) long, of which the first 40 artifact-free epochs were selected. Previous studies in comparable patient populations have investigated functional networks^7^,^8^,^9^,^14^, with results being consistently found in the theta (4–8 Hz), lower alpha (8–10 Hz), and upper alpha (10–13 Hz) bands only. Therefore, band-specific analysis of connectivity and hubs took place only in these bands. BrainWave [CJS, version 0.9.125, http://home.kpn.nl/stam7883/brainwave.html] was used to perform a discrete fast Fourier transform and calculate relative power, as well as the following connectivity analyses. Connectivity between all brain regions was calculated using the phase lag index (PLI), which is relatively insensitive to biases related to neurophysiological signal analysis^26^. This yielded a connectivity matrix for each epoch in each subject.

### Stationary hub score

**Figure 1:**
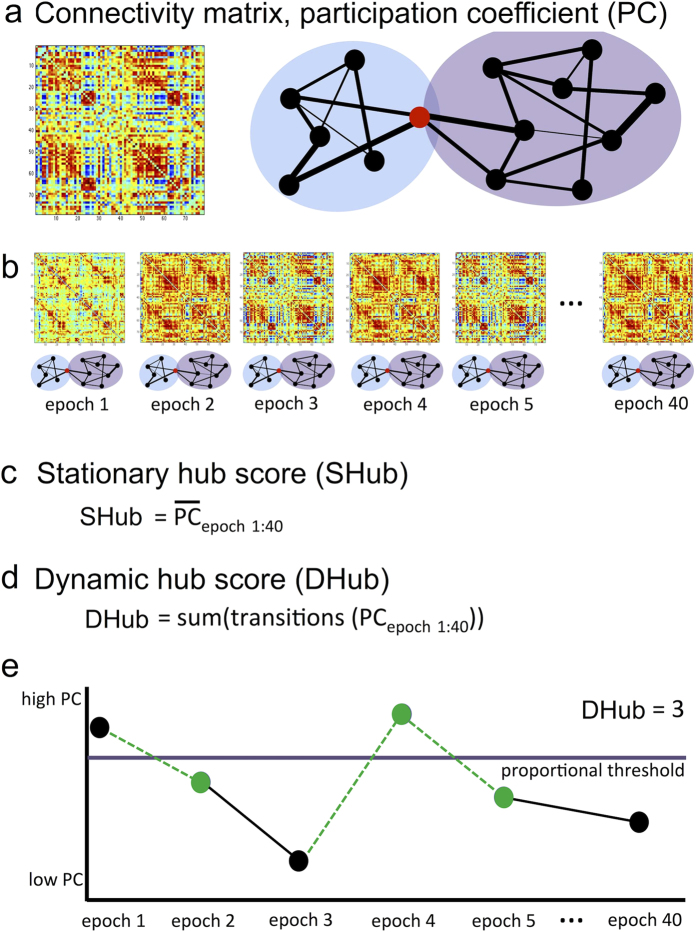
Schematic representation of stationary and dynamic participation coefficient. In (**a**), the left panel shows an example of a weighted connectivity matrix, which forms the basis for the computation of the participation coefficient (PC). Warm and cold colors indicate high and low PLI values, respectively. The nodes are ordered according to Gong and colleagues^43^. The red node in the exemplar network with two modules in the right panel has a high participation coefficient: it has strong connections linking the two modules, compared to its connections within each module. (**b**) indicates our data processing, namely calculation of PC across 40 epochs of MEG data. (**c**) Stationary participation coefficient (SHub) was calculated by averaging PC values of all regions across all 40 epochs. (**d**) Dynamic participation coefficient (DHub) was calculated by summing the number of epochs transitioning across the proportional PC threshold (purple line in **e**), or vice versa. This is further exemplified in (**e**), where three transitions (green dots and dotted lines) across the threshold are present across all depicted epochs, while three nodes/epochs (in black) to not count towards DHub scores. For visualization purposes we only show DHub for a single node.

The analysis pipeline is fully explained in the Supplementary Methods and Fig. 1. In short, stationary hubness per region was operationalized as the extent to which regions connected multiple subsystems or modules of the brain, as previously described^11^,^27^,^28^,^29^,^30^. Stationary hub score (SHub) was then defined as the average of this measure over all epochs and all regions. SHub scores were converted to z-scores based on the healthy MEG control group and were treated as a continuous variable.

In order to be able to control for the possible associations of simpler network measures with cognitive functioning, we also calculated average modularity per subject. This measure indicates the extent to which the brain network is organized into separate communities^28^.

### Dynamic hub score

Dynamic hub score (DHub) assesses the number of transitions between high and low SHub across all epochs and regions. In other words, it measures how often each region switches from high load (hub) to low load (non-hub). In determining the number of transitions, a proportional threshold was used to define high and low load, namely the regions with the top 30% SHub scores versus the bottom 70% SHub scores, respectively. A DHub z-score was calculated for each frequency band per patient using healthy controls as reference. Although the use of a threshold is subjective, this method ensures normalization for overall level of hubness per subject. Also, it allows for possible future use in individual patients without need for a reference. In order to confirm the robustness of our findings, we also tested other proportional thresholds, ranging between 10–40% with 5% increments.

In addition, we calculated the variance in modularity over the within-subject epochs, to control for variation in this more basic measure as a confounder in our analysis of the association between DHub and cognition.

### Statistical analysis

Statistical analyses were performed using IBM SPSS Statistics package version 20.0 (Armonk (NY), USA) and Matlab version r2012b (Natick (MA), USA). Tests for normality (Kolmogorov-Smirnov) were used to check whether parametric testing could be used on SHub and DHub z-scores, which proved to be the case (p > 0.2). Student’s t-tests for independent samples were used to test differences in band-specific relative power between patients at PRE and healthy controls.

Group differences and changes over time with respect to cognitive performance, SHub, and DHub were tested using generalized linear models for repeated measures, using time point and frequency band as within-subject variables and group as between-subject variable.

Associations between SHub, DHub and cognitive functioning at PRE were tested using forward step-wise linear regression, using cognitive functioning as the dependent, and SHub and DHub as covariates together with several patient characteristics (criterion for entry was F probability >0.05 for all forward analyses): type of lesion, diffuse growing pattern according to a histopathologist, temporal versus extratemporal localization, lesion lateralization, handedness, hippocampal atrophy, and lesion volume. A forward regression approach was used to avoid issues with possible collinearity of the SHub and DHub measures and to limit the number of tests used. We report significant results with and without Bonferroni correction for the three analyses performed.

Correlations between changes in cognitive functioning per domain and changes in SHub and DHub were tested using forward linear regression analysis, with forced entry of several known predictors of cognitive outcome (i.e. preoperative verbal memory, presence of hippocampal atrophy, lesion lateralization, seizure-freedom at POST, resection volume, and handedness^1^,^2^) and stepwise entering of the other covariates (lesion volume, extent of resection, type of lesion, and temporal versus non-temporal lesion localization). Again, we report significant results with and without Bonferroni correction for the three analyses performed.

Prediction of cognitive deterioration with preoperative (PRE) SHub and DHub was tested using the continuous cognitive change score by forward linear regression with both forced and stepwise entry of covariates, as described above.

For each of these main analyses, post-hoc exploration of regional SHub and DHub values was performed in the case of significant results in the main regression analyses. In these cases, correlation coefficients between hub scores and cognitive performance or decline were calculated for each region over all patients. In order to facilitate visual assessment of results, figures include subnetwork belonging of each region according to Yeo and colleagues^31^. These subnetworks included the visual network, sensorimotor network, default mode network, limbic network, frontoparietal network, and attention network. However, subnetwork indices were in no way used for the analyses. Furthermore, since these analyses lack statistical power to support any definite conclusions, we supply these figures for further interpretation by the readers.

We also performed a number of control analyses to ascertain that our findings were specific to SHub and/or DHub, instead of also being explained by ‘simple’ functional connectivity, variance in modularity over all epochs, the sum of the change in number of modules across epochs, or the spatial variance in modular partitions over epochs. The latter was operationalized in two ways: firstly, we determined the Dice coefficient of spatial overlap between each modular partition for each separate epoch in each subject^32^, and averaged these values to obtain a single measure of spatial modular stability. Secondly, we calculated the standard deviation of these values over all epochs, to scope spatial modular variability.

Statistical significance was set at p < 0.05, using two-tailed tests. For relevant statistics, 95% confidence intervals (CI) were reported.

## Results

### Subject characteristics

**Figure 2:**
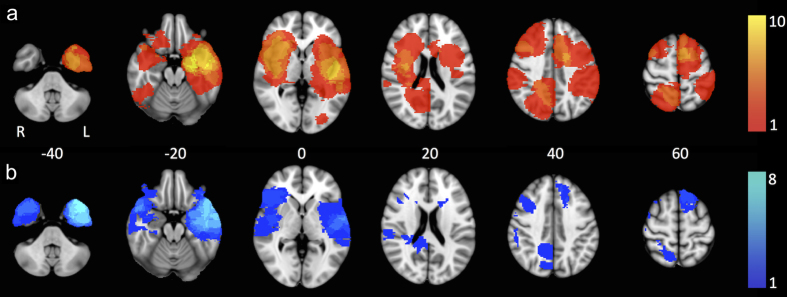
Summed lesion and resection map. Maps are shown at six axial slice locations projected onto the Montreal Neurological Institute brain template, with z-coordinates indicated in the middle row. In (**a**), presurgical lesions were manually drawn in native space per patient, after which images were coregistered with the MNI template. A sum score was obtained by adding all patient lesion maps, with a maximum overlap of lesions occurring in 10 patients. In (**b**), the same procedure was followed for depiction of resection cavities. Here, maximally 8 patients showed overlap of resection locations.

In total, 44 patients were eligible for participation in this study, but 16 patients were excluded due to MEG artifacts or incomplete neuropsychological testing at either PRE or POST, leaving 28 patients in our cohort (Table 1). Most patients (n = 21) had a glioma, while other lesion types included MTS (n = 4), cavernoma (n = 1), or cavernous hemangioma (n = 2). Of the glioma patients, 8 had an astrocytoma (7 diffuse infiltrating), 10 oligodendroglioma, 2 pilocytic astrocytoma (WHO grade I), and 1 ganglioglioma according to histopathological reports. Lesion and resection heat maps can be found in Fig. 2.

Patients had significantly higher relative power in the theta (t(54) = 2.24, 95% CI of difference [0.003–0.047], p = 0.029) and lower alpha bands (t(54) = 2.19, CI [0.002–0.039], p = 0.033) than controls, whereas the upper alpha band did not show significant differences (t(54) = −1.66, CI [−0.031−0.003], p = 0.104).

Patients’ cognitive performance did not change significantly between PRE and POST at the group level (Supplementary Table S1). There were no main or interaction effects of group or time point with respect to SHub and DHub.

### Associations between SHub, DHub, and cognitive functioning at PRE

Executive functioning was significantly related to SHub in the lower alpha band, with higher SHub correlating with poorer performance (p = 0.022, Table 2). The same negative association was present between lower alpha SHub and attention (p = 0.027). However, these associations between SHub and cognitive functioning did not survive Bonferroni correction for the three tests performed (one for each cognitive domain). There was no significant association between cognitive functioning and DHub at PRE (Supplementary Table S2).

### DHub tracks verbal memory post-operatively

**Figure 3:**
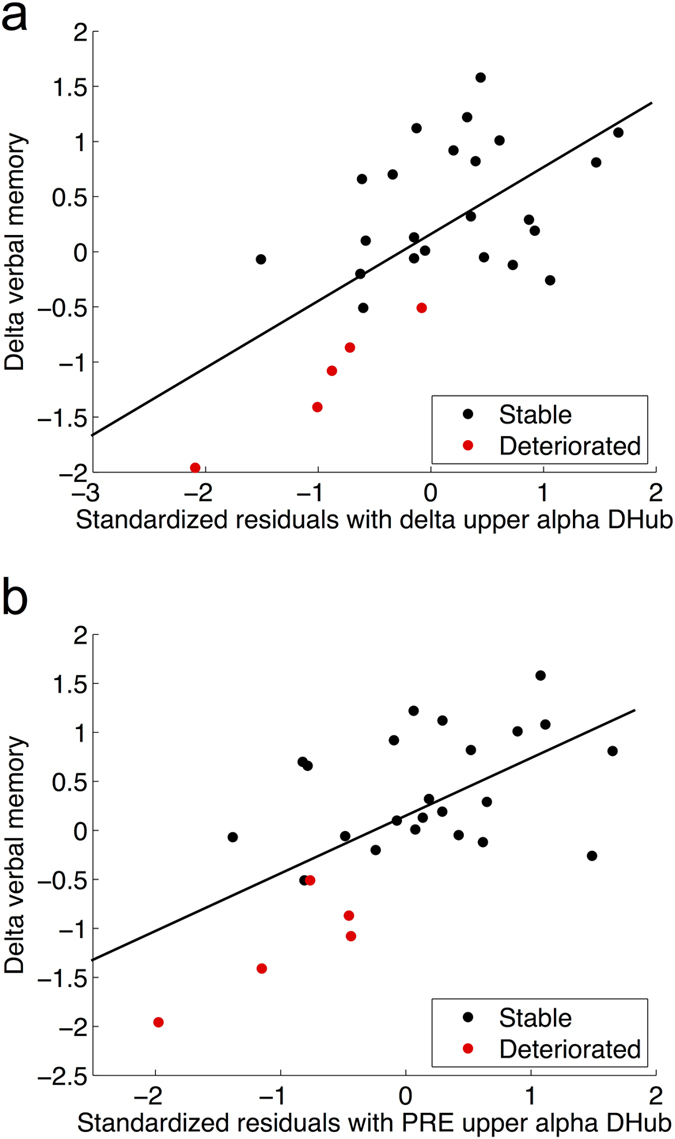
Associations between DHub and cognitive change. (**a**) depicts the association between delta score of upper alpha DHub between the preoperative and 1-year post resection time points and delta score of verbal memory (VM) z-score. Since several clinical variables were also entered into the model, standardized regression residuals are used on the x-axis. In (**b**), the association between preoperative upper alpha band DHub standardized regression residuals and verbal memory outcome is shown.

We then tested whether individual changes or delta scores in cognitive z-score between PRE and POST (POST minus PRE score) could be tracked by SHub or DHub changes (POST minus PRE score). Indeed, delta verbal memory was significantly predicted by the delta score of upper alpha DHub (model p = 0.005, DHub p = 0.015, Table 2 and Fig. 3a), with its addition to the model doubling the explained variance from 24% to 48%. These results survived Bonferroni correction for multiple testing.

Control analyses showed that changes in verbal memory deterioration were not related to changes in average connectivity, variance of modularity, changing number of modules, or the stability or variability of spatial modular partitions. Furthermore, changes in executive functioning and attention were not significantly related to changes in DHub or SHub.

**Figure 4:**
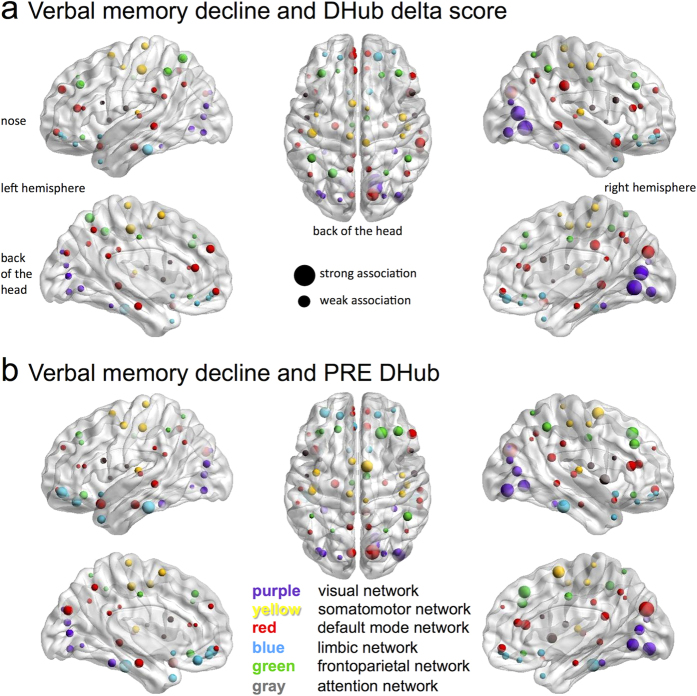
Post-hoc regional analysis of the association between verbal memory and DHub. (**a**) shows the associations between delta score of regional upper alpha DHub and verbal memory change. Each of 78 cortical brain regions is represented by a sphere, with larger spheres indicating higher associations of DHub with verbal memory. Sphere colors indicate subnetworks as defined in Yeo and colleagues^31^. In (**b**), the associations between regional PRE DHub en verbal memory are depicted.

Post-hoc investigations of the spatial location of those regions most predictive of verbal memory decline in terms of delta DHub score were performed within the patients. As can be seen in Fig. 4, particularly DHub changes in the occipital/visual regions (in purple) as well as the regions belonging to the default mode network (in red) were related to cognitive decline. These results may suggest that changes in DHub score of particular subnetworks specifically are indicative of verbal memory decline in the individual network.

### DHub pre-operatively predicts decline in verbal memory

A forward linear regression model showed no significant results regarding the prediction of attention and executive functioning delta scores with PRE SHub or DHub (Supplementary Table S2). However, verbal memory deterioration was significantly predicted by a model incorporating upper alpha DHub at PRE (model p = 0.003, DHub p = 0.010, see Table 2 and Fig. 3b), which remained significant after correction for multiple tests. Control analyses of the association between ‘simpler’ network measures and cognitive decline show that average functional connectivity, variance of modularity, change in number of modules, and spatial modular stability and variability were not significant.

Post-hoc regional analyses were again performed (see Fig. 4), showing that DHub at PRE of particularly the occipital (purple), default mode (red) and frontoparietal (green) regions were predictive of verbal memory decline. There was a significant correlation between the previously mentioned regional delta scores of DHub and PRE scores of DHub related to verbal memory (rho = −0.663, p < 0.001), indicating that indeed, the same regions that were tracking decline were predictors at the individual level.

In order to obtain an indication of predictive accuracy using a post-hoc statistical approach, verbal memory delta scores were dichotomized into stable (<0.5 SD decrease between PRE and POST within each patient) versus deteriorated (>0.5 SD decrease; Supplementary Table S3). Using a logistic regression analysis with this dichotomous variable as the dependent variable and the previously described covariates yielded an overall accuracy of 82%. The model including upper alpha DHub yielded significantly higher accuracy, namely of 89% (model chi-square = 10.46, p = 0.001, beta [95% CI] = 0.052[0.004–0.735], p = 0.029, sensitivity 60%, specificity 96%, significance of change p = 0.001).

### Other DHub thresholds yield similar results

Our results with respect to the predictive value of upper alpha DHub at PRE predicting verbal memory deterioration were replicated using other proportional thresholds. Significant predictive values were found for thresholds from 20 to 40% (p ≤ 0.02; Supplementary Table S4), indicating the robustness of our findings across different thresholds.

## Discussion

Higher stationary hub load in the lower alpha band related to poorer performance in the domains of attention and executive functioning, although these results did not survive corrections for multiple comparisons. Previous work has reported positive associations between hubness and cognition, albeit only in a small cohort of high-grade tumor patients, who were rare in our sample^8^. A certain level of stationary hubness within the resting brain network seems to be a prerequisite for adequate information processing^33^,^34^. However, it may be speculated that overuse of hubs may lead to network damage and consequent cognitive impairment^12^,^35^, although our results do not support a significant role of stationary hub load in this process.

More interestingly, changes in verbal memory correlated positively with changes in DHub, even after correction for multiple comparisons and in comparison to more simple network measures. These findings indicate that adaptation of the network to more dynamic hub load after resection correlates with improved cognitive functioning. These results are in line with cross-sectional studies reporting positively correlated dynamics with cognitive functioning in both healthy subjects and MTS patients^20^,^21^. Furthermore, they support the hypothesized relation between hub (over)load and cognitive deficits.

Even more important, this positive association between dynamics and cognition was not only present for change scores: postoperative verbal memory change was related to preoperative DHub. Specifically, more dynamic hub load before resection was predictive of improving verbal memory. More dynamic hubness preoperatively may be a sign of increased neural and/or cognitive reserve during the postoperative adaptive phase. Comparable results have been reported using transcranial direct current stimulation (tDCS), indicating that intervention effects are largely determined by pre-stimulation network properties^36^,^37^. Moreover, prediction of cognitive outcome with dynamic networks has been reported in a memory task-based longitudinal fMRI study in healthy subjects^38^: increased dynamic hubness during the learning phase was predictive of superior performance during the concluding scanning session.

Exploratory post-hoc regional analyses of DHub, both delta and PRE scores, show the same regions to be associated with verbal memory decline. These areas include parts of the occipital/visual network, the default mode network, and the frontoparietal network. Although these results should be interpreted cautiously due to their explorative nature, previous studies within the same patient population have shown postsurgical alterations in connectivity within the default mode and frontoparietal networks to be related to cognition^10^. Future studies should be aimed at integration of subnetwork features in this dynamic network-based framework of cognitive decline.

In moving towards clinical applications, an open question is whether it is possible to define an exact preoperative DHub cut-off to predict cognitive deterioration. The positive linear relation between preoperative DHub and verbal memory change score suggests it is, but one must keep in mind the relatively limited sample size of this study. Furthermore, our post-hoc logistic regression analysis yielded excellent sensitivity and specificity, but one must keep in mind that these analyses purely serve as an indication of statistical relevance of our findings. In order to conclude anything about clinical use of DHub as a predictor, extensive additional validation is necessary. Another possible shortcoming in our study is the heterogeneous sample. Our cohort may have predominantly been atypical with respect to the risk for postsurgical verbal memory problems, since most patients had extratemporal tumors. Furthermore, the level of diffuse growth of lesion investigated (i.e. ranging from MTS to diffusely infiltrating astrocytoma) may relate to the focal nature of these lesions. From Fig. 2, it is clear that most lesions were indeed in the temporal lobes, while the extratemporal lesions occured in many different locations. It is therefore not expected that the associations we found between cognitive functioning and network characteristics were confounded by particular lesion localization, but with the current group size we lack the statistical power to determine the significance of detailed lesion location on our results. However, in light of the complete lack of prognostics regarding cognitive outcome in the individual patient so far, particularly in these atypical patients, our findings may be all the more interesting.

Our results occurred in the lower and upper alpha bands, which is in line with previous MEG source space studies^9^,^10^. In these studies, theta band correlations of resting-state connectivity with cognitive functioning have also been reported. However, they were based on signal-space MEG work instead of source-localized activity, which means that a large part of preprocessing used in the current study was absent, which may have influenced band-specific findings^8^. We did find an association between resting-state theta band functional connectivity and executive functioning, but not with SHub or DHub. The selection of these three frequency bands was based on this previous literature, which shows most consistent alterations in the theta and alpha bands. Previously posited explanations for this band specificity in lesional epilepsy could be the involvement of GABA-ergic neurotransmission, which is particularly related to theta band activity^39^,^40^. As for the alpha band, this frequency band contains most of the signal measured during eyes-closed resting-state, and may therefore dominate with respect to signal-to-noise ratio and subsequently sensitivity to correlations with functioning. However, it cannot be excluded that associations with (dynamic) hub overload in the delta, beta, and gamma bands may have been missed because of our a priori selection of frequency-bands of interest. Furthermore, we investigated the connectivity correlates of cognitive functioning during resting-state MEG recordings. A growing body of literature suggests that resting-state connectivity patterns are indeed able to capture an important amount of variation in cognitive performance, particularly in this population^41^.

Since DHub is a new measure, we assessed whether different proportional thresholds would yield similar results, which proved to be the case. It would be interesting to further investigate the behavior of DHub over an even larger range of parameter settings and methods (although literature suggests that their influence may be limited^42^), and also in comparison with other recently proposed measures of dynamic connectivity^16^. However, these methodological questions were beyond the scope of this study.

In conclusion, dynamic hub load relates to cognitive functioning in patients undergoing lesion resection, with a particularly interesting predictive value of lower preoperative dynamic hubness for postoperative verbal memory decline. These results support the hypothesis that a loss of dynamic hub load is deleterious for cognitive functioning.

## Additional Information

**How to cite this article**: Carbo, E. W. S. *et al*. Dynamic hub load predicts cognitive decline after resective neurosurgery. *Sci. Rep.* **7**, 42117; doi: 10.1038/srep42117 (2017).

**Publisher's note:** Springer Nature remains neutral with regard to jurisdictional claims in published maps and institutional affiliations.

## Supplementary Material

Supplementary Material

## Figures and Tables

**Table 1:** Subject characteristics.

Variable	Patients (n = 28)	HC (n = 28)
Mean age at PRE in years (SD)	37 (10)	42 (9)
Males (females)	22 (6)	22 (6)
Median education	5	5
Mean relative power at PRE theta (SD)/lower alpha (SD)/upper alpha (SD)	0.20 (0.05)*/0.11 (0.04)*/0.12 (0.03)	0.18 (0.03)/0.09 (0.03)/0.14 (0.03)
Hand preference: right (left)	20 (8)	NA
PRE-surgery interval in months (SD)	3 (4)	NA
Surgery-POST interval in months (SD)	11 (2)	NA
Disease duration at PRE in months (SD)	103 (155)	NA
Type of seizures: partial/complex partial/generalized/partial and generalized	4/5/9/10	NA
Preoperative monthly seizure frequency (SD)	5 (9)	NA
Lesion type: tumor grade I/grade II/grade III (non-tumor)	3/15/3 (7)	NA
Lesion growing pattern: diffuse (non-diffuse)	7 (21)	NA
Lesion lateralization: left (right)	17 (11)	NA
Lesion location: temporal (extratemporal)	16 (12)	NA
Lesion volume in cm^3^ (SD)	31 (29)	NA
Hippocampus: intact (sclerotic)	24 (4)	NA
Resection volume in cm^3^ (SD)	39 (25)	NA
Gross total resection (subtotal)	20 (8)	NA
Seizure free at POST (not seizure free)	21 (7)	NA

*p < 0.05 difference between patients and healthy controls. PRE = preoperative time point, POST = 1 year postoperative time point.

**Table 2:** Significant regression analyses predicting cognitive functioning with SHub and DHub.

Dependent	Domain	Model	Adj. R^2^	P-value	Predictors	Beta (95% CI)	P-value
		F (df)			Variables		
PRE cog	A	5.50 (1, 26)	0.143	0.027	PRE lower alpha SHub	−0.418 [−0.856; −0.056]	0.027
	EF	5, 90 (1, 26)	0.154	0.022	PRE lower alpha SHub	−0.430 [−0.969; −0.081]	0.022
Δ cog	VM^¶^	2.71 (5, 22)	0.240	0.047	Hand preference	0.233 [−0.268; 1.11]	0.218
					VM at PRE	−2.94 [−0.926; −0.160]	0.008*
					Hippocampus	0.980 [−0.455; 1.27]	0.338
					Lesion lateralization	1.458 [−0.187; 1.08]	0.159
					Seizure freedom	1.737 [−0.114; 1.29]	0.096
	VM^§^	4.33 (7, 20)	0.463	0.005*	Δ upper alpha DHub	−2.67 [−0.443; −0.055]	0.015*
					Resection volume	−2.33 [−0.022; −0.001]	0.030*
	VM^†^	4.58 (7, 20)	0.481	0.003*	PRE upper alpha DHub	2.844 [0.080; 0.519]	0.010*
					Resection volume	−2.40 [−0.022; −0.002]	0.026*

*p < 0.05 after correction for multiple comparisons, PRE = preoperative time point, A = attention, EF = executive functioning, VM = verbal memory, SHub = stationary hub score, DHub = dynamic hub score, ^¶^indicates prediction model with literature-based predictors, ^§^refers to model using delta score of upper alpha DHub as predictor of delta score of verbal memory in addition to literature-based predictors, ^†^indicates model with preoperative upper alpha DHub as predictor of delta score of verbal memory in addition to literature-based predictors.
