# Supplementary material for: Dynamic hub load predicts cognitive decline after resective neurosurgery
Source: Sci Rep. 2017 Feb 7;7:42117. doi: 10.1038/srep42117 (PMC5294457; doi:10.1038/srep42117)
Supplement: Supplementary Material [file srep42117-s1.pdf]

Dynamic hub load predicts cognitive decline after resective neurosurgery

Ellen W.S. Carbo,[1] Arjan Hillebrand,[2] Edwin van Dellen,[3,4] Prejaas Tewarie,[5,6] Philip C. de Witt Hamer,[7,8] Johannes C. Baayen,[7] Martin Klein,[8,9] Jeroen J.G. Geurts,[1] Jaap C. Reijneveld,[5,8] Cornelis J. Stam,[2] and Linda Douw[1,8,10,*]

[1] Department of Anatomy & Neurosciences, VU University Medical Center, Neuroscience Campus Amsterdam, Amsterdam, The Netherlands

[2] Department of Clinical Neurophysiology and MEG Center, VU University Medical Center, Amsterdam, The Netherlands

[3] Department of Psychiatry, University Medical Center Utrecht, Utrecht, The Netherlands

[4] Brain Center Rudolf Magnus, Utrecht, The Netherlands

[5] Department of Neurology, Neuroscience Campus Amsterdam, VU University Medical Center, Amsterdam, The Netherlands

[6] Sir Peter Mansfield Imaging Centre, school of physics, University of Nottingham, Nottingham

[7] Department of Neurosurgery, Neuroscience Campus Amsterdam, VU University Medical Center, Amsterdam, The Netherlands

[8] VUmc CCA Brain Tumor Center Amsterdam, Amsterdam, The Netherlands

[9] Department of Medical Psychology, VU University Medical Center, Amsterdam, The Netherlands

[10] Athinoula A. Martinos Center for Biomedical Imaging, Massachusetts General Hospital, Charlestown, MA, USA

*Correspondence to l.douw@vumc.nl

Supplementary material

Materials and methods

Neuropsychological assessment

Individual cognitive performance measurement was based on previously reported methods[1–4]. For each patient, a healthy control subject (individually matched for age, sex, and educational level) was derived from a normative sample[5]. Educational level was assessed with an 8-point scale scoring system, ranging from not having finished primary education (level 1) to having obtained a university degree (level 8[6]). A subtest z-score was calculated by comparing each patient's test score with the mean and standard deviation (SD) of each collective test score of the matched healthy controls. The same mean and SD of the control group were used to calculate z-scores for patients' repeated assessments. The subtest z-scores were then averaged into cognitive domains previously reported in comparable patient populations[1,3,4], namely verbal memory (Rey Auditory Verbal Learning Test), attention (Stroop Color Word Test), and executive functioning (Concept Shifting Test and Category Fluency, or Trail Making Test and Letter Fluency).

Magnetoencephalography

Five minutes of MEG data were recorded during an eyes-closed resting-state condition with a sample frequency of 1250 Hz. Anti-aliasing (410 Hz) and high-pass (0.1 Hz) filters were applied online. Malfunctioning channels were identified by visual inspection by three independent raters [EC; EvD; PT] and excluded. The temporal extension of Signal Space Separation (tSSS[7]) was then applied offline with MaxFilter software (Elekta Neuromag Oy, version 2.2.15) to remove noise.

The head position relative to the MEG sensors was recorded continuously using the signals from four head-localization coils. The head-localization coil positions were digitized, as was the outline of the participants' scalp (~500 points), using a 3D digitizer (3 Space Fastrak, Polhemus, Colchester, VT, USA). This scalp surface was used for co-registration with the patient's anatomical T1-weighted MRI, yielding an accuracy of approximately 4mm. The co-registered MRI was spatially normalized to a template MRI using the SEG-toolbox in SPM8[8], to allow for the use of the automated anatomical labeling (AAL) atlas with subjects' normalized co-registered MRI[9], Subcortical structures were removed, as MEG is more sensitive to neural activity in cortical regions, and the voxels in the remaining 78 cortical parcels were used for further analyses.

Time-series estimation

We used a beamformer approach as described before[3,10]. In summary, neuronal activity was reconstructed using a scalar beamformer implementation (Elekta Neuromag Oy, beamformer, version 2.1.28[11]), which sequentially reconstructs activity for each voxel in a predefined grid covering the entire brain (spacing 2mm) by selectively weighting the contribution from each MEG sensor (magnetometers and gradiometers) to a voxel's time-series, after which the voxel with the peak power was chosen representative for the region[10]. For each subject, the first 40 source-reconstructed artifact-free epochs of 2048 samples (1.638s per epoch, 65.5s in total) were selected and band-pass filtered into frequency bands.

Stationary hub score

The next step was to achieve modular decomposition of the network, which was performed on the weighted connectivity matrices as described in detail previously[12–14]. In short, the relation between intra- and intermodular connections determines the strength of each module, with total modularity being optimized. The optimum modular partition was identified separately for each epoch of every person for all the different frequency bands. Then, the participation coefficient (PC) was computed per region, which assesses the strength of connectivity between modules compared to the strength of connectivity within modules, indicating how much relative communication takes place between different subsystems of the brain[15,16]:

$$PC = 1 - \sum_{M} \left(\frac{k\_within}{k\_all} \right)^2$$

where k_within is the sum of all links of the region to all regions within its module, k_all is the sum of links to all other regions, and M signifies the complete set of modules. The average of this measure over all regions and epochs per subject was calculated, yielding a stationary hub score (SHub) for each frequency band. These scores were then converted to a z-score based on the mean and standard deviation of the healthy MEG control group.

Dynamic hub score
We then defined a new method of investigating the dynamics of intermodular connectivity, the specific aim of which was to operationalize the extent of change in hubness of all regions in the brain. Therefore, based on the individual PC for each epoch per subject, the dynamic hub score (DHub) was defined as the number of transitions between high PC and low PC across all epochs and parcels.

Supplementary Table S1. Main effects related to cognitive performance and hub scores. SHub = stationary hub score, DHub = dynamic hub score, PRE = preoperative, POST = 1 year post surgery, HC = healthy controls.

Variable	Effect	F-value (df)	p-value
Cognition	Time (PRE vs POST)	0.989 (1,27)	0.352
	Domain	3.301 (2,26)	0.053
SHub	Frequency band (3 levels)	1.272 (2,53)	0.289
	Time (PRE vs POST)	0.382 (1,54)	0.539
	Group (patients vs HC)	0.313 (1,54)	0.578
DHub	Frequency band (3 levels)	0.314 (2,53)	0.732
	Time (PRE vs POST)	0.636 (1,54)	0.428
	Group (3 levels)	0.839 (1,54)	0.364

Supplementary Table S2. Significant and non-significant predictors of cognitive functioning. sPC = stationary participation coefficient, dPC = dynamic participation coefficient, PRE = presurgical, A = attention, EF = executive functioning, VM = verbal memory, A1 = lower alpha, A2 = upper alpha, n.s. = not significant, delta = change between PRE and POST, hand pref = hand preference, hippo = hippocampus, lat = lateralization, SF = seizure free, res vol = resection volume, * $p < 0.05$, ** $p < 0.01$.

		Attention			
type	cognitive domain	predictor	beta	t-value	p-value
PRE MEG & cogn.	attention	tumor volume	0.185	1.031	0.312
		lesion type	-0.231	-1.306	0.204
		diffuse	0.12	0.612	0.546
		temp loc	0.025	0.138	0.891
		lateralisation	0.006	0.031	0.975
		hippo	-0.335	-1.985	0.058
		hand pref	-0.07	-0.381	0.706
		SF at POST	0.08	0.439	0.664
		T sPC	0.219	1.219	0.234
		A1 sPC	-0.418	-2.345	0.027
		A2 sPC	-0.051	-0.283	0.78
		T dPC	-0.096	-0.488	0.63
		A1 dPC	0.047	0.256	0.8
		A2 dPC	0.12	0.657	0.517
		predictor	beta	t-value	p-value
	executive functioning	tumor volume	0.05	0.273	0.787
		lesion type	-0.119	-0.663	0.513
		diffuse	0.038	0.208	0.837
		temp loc	-0.152	-0.842	0.408
		lateralisation	0.066	0.356	0.725
		hippo	-0.174	-0.984	0.335

		hand pref	0.031	0.168	0.868
		SF at POST	0.182	1.022	0.316
		T sPC	0.204	1.135	0.267
		A1 sPC	-0.43	-2.43	0.022
		A2 sPC	0.062	0.342	0.735
		T dPC	-0.192	-0.998	0.328
		A1 dPC	0.116	0.645	0.525
		A2 dPC	0.043	0.235	0.816
		predictor	beta	t-value	p-value
	verbal memory	tumor volume	0	-0.001	0.999
		lesion type	-0.27	-1.111	0.277
		diffuse	-0.145	-0.594	0.558
		temp loc	-0.233	-1.199	0.242
		lateralisation	0.202	1.026	0.315
		hippo	-0.3	-1.406	0.172
		hand pref	-0.179	-0.903	0.375
		SF at POST	0.281	1.393	0.176
		T sPC	0.172	0.867	0.394
		A1 sPC	-0.103	-0.511	0.614
		A2 sPC	-0.046	-0.223	0.825
		T dPC	-0.282	-1.463	0.156
		A1 dPC	0.251	1.271	0.215
		A2 dPC	0.097	0.466	0.645
Delta MEG & delta cogn.	attention	predictor	beta	t-value	p-value
		tumor volume	-0.24	-0.971	0.342
		lesion type	0.356	1.147	0.264
		diffuse	-0.17	-0.664	0.514

	temp loc	-0.037	-0.147	0.885
	lateralisation	0.134	0.54	0.592
	hippo	0.144	0.647	0.524
	hand pref	0.092	0.4	0.693
	SF at POST	0.002	0.009	0.993
	resection volume	-0.292	-1.328	0.198
	VM at PRE	0.059	0.259	0.798
	delta T sPC	0.21	0.861	0.399
	delta A1 sPC	-0.39	-1.914	0.069
	delta A2 sPC	-0.125	-0.515	0.612
	delta T dPC	-0.059	-0.273	0.788
	delta A1 dPC	0.317	1.406	0.174
	delta A2 dPC	0.164	0.723	0.478
executive functioning	predictor	beta	t-value	p-value
	tumor volume	-0.292	-1.306	0.206
	lesion type	0.089	0.303	0.765
	diffuse	-0.158	-0.673	0.508
	temp loc	0.064	0.28	0.783
	lateralisation	0.002	0.008	0.994
	hippo	0.219	1.064	0.299
	hand pref	-0.335	-1.608	0.122
	SF at POST	0.117	0.565	0.578
	resection volume	-0.381	-1.964	0.063
	VM at PRE	-0.335	-1.608	0.122
	delta T sPC	-0.222	-0.991	0.333
	delta A1 sPC	-0.356	-1.89	0.073
	delta A2 sPC	-0.148	-0.665	0.513

	delta T dPC	-0.328	-1.739	0.097
	delta A1 dPC	0.198	0.927	0.364
	delta A2 dPC	-0.147	-0.7	0.491
verbal memory	predictor	beta	t-value	p-value
	tumor volume	0.12	0.389	0.702
	lesion type	-0.156	-0.671	0.51
	diffuse	0.142	0.751	0.462
	temp loc	0.04	0.216	0.831
	lateralisation	0.298	1.83	0.082
	hippo	0.169	1.128	0.273
	hand pref	0.172	1.037	0.312
	SF at POST	0.399	2.601	0.017*
	resection volume	-0.353	-0.233	0.030*
	VM at PRE	-0.595	-3.886	0.001**
	delta T sPC	-0.119	-0.72	0.481
	delta A1 sPC	-0.24	-1.707	0.104
	delta A2 sPC	-0.244	-1.524	0.144
	delta T dPC	-0.123	-0.84	0.411
	delta A1 dPC	0.124	0.72	0.48
	delta A2 dPC	-0.407	-2.672	0.015*
	delta T PLI	-0.095	-0.583	0.567
	delta A1 PLI	-0.022	-0.134	0.895
	delta A2 PLI	0.088	0.544	0.593
	delta T no modules	0.002	0.015	0.988
	delta A1 no modules	-0.138	-0.816	0.425
	delta A2 no modules	0.127	0.727	0.476

		delta T modularity	-0.035	-0.207	0.838
		delta A1 modularity	0.205	1.292	0.212
		delta A2 modularity	0.057	0.342	0.736
		delta A2 Dice	0.119	0.758	0.457
		delta A2 Dice SD	0.187	1.224	0.236
PRE MEG & delta cogn.	attention	predictor	beta	t-value	p-value
		tumor volume	-0.24	-0.971	0.342
		lesion type	0.356	1.147	0.264
		diffuse	0.064	0.28	0.783
		temp loc	-0.037	-0.147	0.885
		lateralisation	0.124	0.544	0.592
		hippo	0.144	0.647	0.524
		hand pref	0.092	0.4	0.693
		SF at POST	0.002	0.009	0.993
		resection volume	-0.292	-1.328	0.198
		VM at PRE	0.059	0.259	0.798
		T sPC	-0.364	-1.713	0.101
		A1 sPC	0.131	0.597	0.557
		A2 sPC	-0.177	-0.748	0.463
		T dPC	-0.109	-0.481	0.635
		A1 dPC	-0.279	-1.162	0.258
		A2 dPC	-0.029	-0.13	0.898
	executive functioning	predictor	beta	t-value	p-value
		tumor volume	-0.292	-1.306	0.206
		lesion type	0.089	0.303	0.765

	diffuse	-0.17	-0.664	0.514
	temp loc	-0.158	-0.673	0.508
	lateralisation	0.002	0.008	0.994
	hippo	0.219	1.064	0.299
	hand pref	-0.015	-0.072	0.943
	SF at POST	0.117	0.565	0.578
	resection volume	-0.381	-1.964	0.063
	VM at PRE	-0.335	-1.608	0.122
	T sPC	-0.06	-0.289	0.776
	A1 sPC	0.089	0.439	0.665
	A2 sPC	-0.334	-1.602	0.124
	T dPC	-0.082	-0.393	0.698
	A1 dPC	-0.065	-0.284	0.779
	A2 dPC	0.064	0.315	0.756
verbal memory	predictor	beta	t-value	p-value
	tumor volume	0.032	0.101	0.92
	lesion type	-0.177	-0.778	0.446
	diffuse	0.128	0.69	0.498
	temp loc	0.071	0.398	0.695
	lateralisation	0.374	2.362	0.028*
	hippo	0.164	1.109	0.28
	hand pref	0.22	1.388	0.181
	SF at POST	0.338	2.246	0.036*
	resection volume	-0.358	-2.43	0.026*
	VM at PRE	-0.615	-4.068	0.001**
	T sPC	0.001	0.006	0.995
	A1 sPC	0.179	1.156	0.262
	A2 sPC	-0.077	-0.439	0.666

T dPC	0.101	0.665	0.514
A1 dPC	-0.044	-0.244	0.81
A2 dPC	0.41	2.844	0.010*
T PLI	0.093	0.489	0.631
A1 PLI	0.015	0.096	0.925
A2 PLI	0.041	0.266	0.793
T no modules	-0.311	-1.771	0.093
A1 no modules	0.25	1.482	0.155
A2 no modules	-0.086	-0.501	0.622
T modularity	-0.089	-0.573	0.574
A1 modularity	0.005	0.031	0.975
A2 modularity	0.122	0.805	0.431
A2 Dice	-0.155	-0.861	0.4
A2 Dice SD	-0.275	-1.848	0.08

Supplementary Table S3. Patient characteristics of patients with stable versus deteriorated verbal memory. PRE = preoperative time point, POST = postoperative time point. Test statistic indicates chi-square for categorical and dichotomous variables, Mann-Whitney U for continuous variables. All p-values are exact and two-tailed.

Variable	Stable (n=23)	Deteriorated (n=5)	Test stat	p-value
Mean age at PRE in years (SD)	38 (10)	35 (9)	47	0.560
Males (females)	17 (6)	5 (0)	1.660	0.553
Median education	5	4	4.399	0.531
Hand preference: right (left)	15 (8)	5 (0)	2.435	0.281
Disease duration at PRE in months (SD)	113 (170)	57 (36)	80	0.193
PRE monthly seizure frequency (SD)	5 (9)	2 (3)	66	0.641
Lesion type: tumor (non-tumor)	17 (6)	4 (1)	0.081	0.999
Lesion lateralization: left (right)	14 (9)	3 (2)	0.001	0.999
Lesion location: temporal (extratemporal)	15 (8)	4 (1)	3.429	0.133
Lesion volume in cm^3 (SD)	33 (30)	27 (23)	50	0.684
Hippocampus: intact (sclerotic)	19 (4)	5 (0)	1.014	0.999
Resection volume in cm^3 (SD)	40 (27)	36 (19)	55	0.908
Gross total resection (subtotal)	16 (7)	4 (1)	0.219	0.999
Seizure free at POST (not seizure free)	16 (7)	5 (0)	2.029	0.290

Supplementary table S4. Replication of verbal memory outcome results with different DHub proportional thresholds. * p<0.05, ** p<0.01, DHub = dynamic hub score, SHub = stationary hub score.

Threshold	Model chi-square (df)	p-value
10% highest SHub values	0.01 (1)	0.998
15% highest SHub values	2.40 (1)	0.122
20% highest SHub values	5.85 (1)	0.016*
25% highest SHub values	8.55 (1)	0.003**
Original threshold (30%)	10.5 (1)	0.001**
35% highest SHub values	8.53 (1)	0.004**
40% highest SHub values	5.45 (1)	0.020*

References

1. Douw, L. *et al.* Cognitive and radiological effects of radiotherapy in patients with low-grade glioma: long-term follow-up. *Lancet Neurol* **8,** 810–818 (2009).
2. van Dellen, E. *et al.* Connectivity in MEG resting-state networks increases after resective surgery for low-grade glioma and correlates with improved cognitive performance. *NeuroImage. Clin.* **2,** 1–7 (2012).
3. van Dellen, E. *et al.* Epilepsy surgery outcome and functional network alterations in longitudinal MEG: a minimum spanning tree analysis. *Neuroimage* **86,** 354–63 (2014).
4. Douw, L. *et al.* Cognition is related to resting-state small-world network topology: an magnetoencephalographic study. *Neuroscience* **175,** 169–177 (2011).
5. Jolles, J., Houx, P. J., van Boxtel, M. P. & Ponds, R. W. H. M. *Maastricht Aging Study: determinants of cognitive aging*. (Neuropsych Publishers, 1995).
6. Verhage, F. Intelligence and Age in a Dutch Sample. *Hum. Dev.* **8,** 238–245 (1965).
7. Taulu, S. & Hari, R. Removal of magnetoencephalographic artifacts with temporal signal-space separation: demonstration with single-trial auditory-evoked responses. *Hum. Brain Mapp.* **30,** 1524–34 (2009).
8. Weiskopf, N. *et al.* Unified segmentation based correction of R1 brain maps for RF transmit field inhomogeneities (UNICORT). *Neuroimage* **54,** 2116–24 (2011).
9. Tzourio-Mazoyer, N. *et al.* Automated anatomical labeling of activations in SPM using a macroscopic anatomical parcellation of the MNI MRI single-subject brain. *Neuroimage* **15,** 273–89 (2002).
10. Hillebrand, A., Barnes, G. R., Bosboom, J. L., Berendse, H. W. & Stam, C. J. Frequency-dependent functional connectivity within resting-state networks: an atlas-based MEG beamformer solution. *Neuroimage* **59,** 3909–21 (2012).
11. Robinson, S. E. & Vrba, J. in *Recent advances in biomagnetism* (eds. Yoshimoto, T., Kotani, M., Kuriki, S., Karibe, H. & Nakasato, N.) 302–305 (Tohoku University Press, 1999).
12. Stam, C. J., Hillebrand, A. J., Wang, H. & van Mieghem, P. Emergence of modular structure in a large-scale brain network with interactions between dynamics and connectivity. *Front Comput Neurosci* **4,** 133 (2010).
13. de Haan, W., Mott, K., van Straaten, E. C. W., Scheltens, P. & Stam, C. J. Activity dependent degeneration explains hub vulnerability in Alzheimer's disease. *PLoS Comput. Biol.* **8,** e1002582 (2012).
14. Newman, M. E. Analysis of weighted networks. *Phys Rev E Stat Nonlin Soft Matter Phys* **70,** 56131 (2004).
15. Guimera, R. & Nunes Amaral, L. A. Functional cartography of complex metabolic networks. *Nature* **433,** 895–900 (2005).
16. de Haan, W. *et al.* Disrupted modular brain dynamics reflect cognitive dysfunction in Alzheimer's disease. *Neuroimage* **59,** 3085–93 (2012).
